# Musculoskeletal Pain as a Risk Factor for Poor Dizziness Outcomes: A Longitudinal Study Among Patients With Persistent Vestibular Dizziness

**DOI:** 10.1093/ptj/pzaf001

**Published:** 2025-01-13

**Authors:** Unni Moen, Roy Miodini Nilsen, Mari Kalland Knapstad, Kjersti Thulin Wilhelmsen, Stein Helge Glad Nordahl, Frederik Kragerud Goplen, Dara Meldrum, Liv Heide Magnussen

**Affiliations:** Department of Health and Functioning, Western Norway University of Applied Sciences, 5063 Bergen, Norway; Department of Health and Functioning, Western Norway University of Applied Sciences, 5063 Bergen, Norway; Department of Health and Functioning, Western Norway University of Applied Sciences, 5063 Bergen, Norway; Norwegian National Network for Vestibular Disorders, Department of Otorhinolaryngology & Head and Neck Surgery, Haukeland University Hospital, 5021 Bergen, Norway; Department of Health and Functioning, Western Norway University of Applied Sciences, 5063 Bergen, Norway; Norwegian National Network for Vestibular Disorders, Department of Otorhinolaryngology & Head and Neck Surgery, Haukeland University Hospital, 5021 Bergen, Norway; Department of Clinical Medicine, University of Bergen, Klinisk institutt 1, 5021 Bergen, Norway; Norwegian National Network for Vestibular Disorders, Department of Otorhinolaryngology & Head and Neck Surgery, Haukeland University Hospital, 5021 Bergen, Norway; School of Medicine, Trinity College Dublin, Dublin 2, D02PN40 Dublin, Ireland; Department of Health and Functioning, Western Norway University of Applied Sciences, 5063 Bergen, Norway

**Keywords:** Adult, Balance, Chronic Pain, Dizziness, Musculoskeletal Pain, Pain, Vestibular System, Vestibular Diseases, Vertigo

## Abstract

**Objective:**

Musculoskeletal pain and psychological distress are prevalent comorbidities in patients with persistent dizziness. Little is known about how comorbid pain influences the outcome of persistent dizziness. This study examined the impact of pain on dizziness outcomes and the potential modifying role of psychological distress.

**Methods:**

This study was a longitudinal study of 150 patients with persistent dizziness. Vertigo Symptom Scale - short form (VSS-SF), Dizziness Handicap Inventory (DHI), number of pain sites, pain intensity, and Hospital Anxiety and Depression Scale (HADS) were assessed at baseline, 6 months and 12 months. Linear mixed effects model for longitudinal data was used to explore the association between musculoskeletal pain and dizziness. Interaction analysis was used to assess whether psychological distress had a modifying effect on the association between pain and dizziness.

**Results:**

VSS-SF and DHI decreased during follow-up but not to a clinically relevant level for the patients. Patients reporting comorbid psychological distress reported higher scores on VSS-SF, DHI, more pain sites and higher pain intensity. A positive association was observed between the number of pain sites and VSS-SF and between pain intensity and VSS-SF, and these associations were stronger in patients reporting psychological distress. Similar associations were found for DHI. Patients reporting ≥4 pain sites or pain intensity of ≥4 out of 10 at baseline, still reported severe dizziness and moderate disability 12 months later.

**Conclusion:**

Musculoskeletal pain is a risk factor for poor dizziness outcomes, especially when comorbid psychological distress is present. Clinicians should be attentive to musculoskeletal pain when the number of pain sites exceeds 4 or pain intensity exceeds 4 on a numeric rating scale.

**Impact:**

A new understanding of the impact of musculoskeletal pain on persistent dizziness could be the key to successful recovery and the prevention of prolonged issues.

## INTRODUCTION

Dizziness is a common complaint with a wide range of underlying causes. Among these, disorders affecting the vestibular system are common.^1–3^ Nonetheless, it is important to note that the ultimate cause of dizziness is not always identified.^1^ Dizziness is linked to a multitude of negative consequences such as psychological comorbidity, reduced quality of life,^4^^,^^5^ as well as financial burdens^6^^,^^7^ affecting individual patients and the health care system. Following an acute vestibular onset, symptoms often recede within a short time, yet in 25% to 50%, symptoms are prolonged.^8–11^ In contrast to the functional status of the vestibular system, coexisting factors such as anxiety and depression, sex^4^^,^^12–14^ and specific personality traits^12^^,^^15^^,^^16^ appear to have a role in the process. Psychiatric comorbidity is highly prevalent,^4^ and anxiety is the most common second diagnosis^17^ in patients with dizziness. The fear of triggering dizziness and the potential risk of falls may lead to restricted movement patterns which over time could result in increased muscular tension.^18^^,^^19^ This maladaptive response can contribute to musculoskeletal complaints. However, musculoskeletal pain has received little attention in the literature on patients with persistent dizziness. The exception is cervical dizziness, where a relationship between neck pain and dizziness has frequently been reported.^20–22^ A systematic review of the prevalence of musculoskeletal pain in patients with dizziness revealed high occurrence, not only in the neck but also in other body regions.^23^ Further, a recent cross-sectional study involving patients with persistent dizziness (≥ 3 months) found similar results regarding widespread pain patterns and also associations between dizziness and pain.^24^ This latter study also established that dizziness severity was associated with a higher number of pain sites and pain intensity, while dizziness-related disability was associated with the number of pain sites only. The high proportion of individuals developing persistent symptoms of dizziness supports the need to explore possible sustainable factors and potential obstacles to successful recovery. Enhancing our understanding of how comorbid musculoskeletal pain could impact dizziness-related outcomes among these patients over time, may guide targeted treatment for patients with persistent dizziness. We hypothesized that individuals with higher levels of comorbid musculoskeletal pain would also have a sustained higher degree of dizziness and greater disability during follow-up. We also hypothesized that the presence of psychological distress would further amplify this relationship.

Therefore, the present study aimed to investigate whether comorbid musculoskeletal pain could be a risk factor for poorer dizziness-related outcomes at 6-month and 12-month follow-ups in patients with persistent dizziness and whether psychological distress could have a modifying effect on the association between pain and dizziness over time.

## METHODS

### Design and Settings

In this longitudinal prospective cohort study, 150 patients with persistent (≥3 months) dizziness of suspected vestibular origin were consecutively recruited from a specialized balance clinic within a Norwegian university hospital. Data collection at baseline occurred at the patients’ scheduled appointments between August 2020 and January 2022. Demographic information and information related to dizziness symptoms, psychological distress and pain were collected through standardized questionnaires. Six and 12 months after entering the study, the patients received the same questionnaires either online or, if preferred, in paper format by post. The online data collection was administered using the SurveyXact web application, which adheres to the General Data Protection Regulation (GDPR) and International Standards of Web Accessibility (WCAG 2.1). The survey email was supported by an informal SMS as a reminder and to avoid the survey being sorted as spam. Another reminder was dispatched approximately 10 days later. The study design is depicted in Supplementary Figure 1.

### Participants

Patients aged 18 to 67 were invited to participate and included if their dizziness lasted ≥3 months. The patients were referred from primary care or other specialists to the Balance Clinic due to constant or recurrent symptoms of dizziness of suspected vestibular origin. The common consensus for defining persistent symptoms is 3 months or longer.^9^ Hospitalized patients, patients diagnosed with vestibular schwannomas, divers investigated for neuro-otologic disorder, and patients with severe comorbidities that could potentially impact their physical functioning (such as multiple sclerosis, amputation, and alcoholism) were excluded. As the clinic is a tertiary clinic for second opinions and special cases, patients from other health regions were excluded to avoid overrepresentation of such cases. The participants signed a written consent form, which included the option to withdraw at any time without providing a reason. Sufficient knowledge of Norwegian to fill in the questionnaires was also required.

### Data Collection

#### Demographic Variables

Participants’ age, sex, diagnosis, and duration of dizziness were registered. All patients were examined and diagnosed and further categorized into *episodic* (eg, benign paroxysmal positional vertigo [BPPV], vestibular migraine, Ménière disease), *chronic* (eg, persistent postural-perceptual dizziness [PPPD]), and other *non-vestibular* diagnoses (eg, panic attack) according to the ICD-11 criteria.^25^ The clinical examination included pure-tone audiometry, dynamic posturography, videonystagmography with measurements of ocular smooth pursuit, saccades, and bithermal caloric tests, a standard ear, nose, and throat (ENT) examination, and a physical therapy consultation. During the 6-month and 12-month follow-up, responders were asked if they had experienced dizziness in the previous 6 months.

#### Outcomes

The Dizziness Handicap Inventory (DHI) is a 25-item questionnaire developed to assess the impact of dizziness on daily life.^26^ The questionnaire reflects physical, emotional, and functional aspects of dizziness with response categories “yes” (4 points), “sometimes” (2 points), and “no” (0 points). Scores ≤30 (out of 100) indicate mild, 31 to 60 moderate, and > 60 a severe dizziness disability.^27^ The Norwegian translation of DHI has demonstrated satisfactory measurement properties as a discriminate and evaluative tool.^28^

The Vertigo Symptom Scale – short form (VSS-SF) is a 15-item questionnaire developed to assess the frequency and severity of dizziness symptoms. Each item is scored on a 5-point scale, from 0 (“never”) to 4 (“very often [almost every day]”). A score ≥ 12 (out of 60) points indicates severe dizziness.^29^ The Norwegian translation of VSS-SF has shown satisfactory construct validity, internal consistency, discriminate ability, and test–retest reliability.^30^

#### Exposures

The Standardized Nordic Pain Questionnaire (SNQ) was used to identify the localization of pain or discomfort in the body. In a mannequin drawing, the body is divided into 10 regions: head, neck, shoulders, elbows, wrist/hands, upper back, lower back, hips, knees, ankle/feet. Patients indicated pain or discomfort in any of the regions during the last 7 days. The number of pain sites (NPS) was recorded. NPS is strongly associated with reduced overall health, sleep quality, and psychological health.^31^^,^^32^

Pain intensity was registered by a numeric rating scale (NRS) ranging from 0 to 10, where a score of 0 equals “no pain at all” and 10 equals “worst imaginable pain”. Pain intensity scores between 1 to 3 are categorized as mild, 4 to 6 as moderate, and 7 to 10 as severe pain.^33^ NRS is an easy, valid, and reliable tool for measuring pain.^34^

#### Effect Modifier

The Hospital Anxiety and Depression Scale (HADS) is a 14-item questionnaire assessing psychological distress. It comprises an anxiety and a depression subscale, each containing 7 questions with score options from 0 to 3. The most commonly used categorization of HADS scores on the anxiety and depression subscales is 0 to 7, indicating non-cases, 8 to 10 mild, 11 to 14 moderate, and 15 to 21 severe symptoms.^35^ A Norwegian population study suggests a cut-off score of ≥19 on the total scale, indicating psychological distress.^36^

### Statistical Analysis

This study used linear mixed effects models to investigate the relationship between musculoskeletal pain and dizziness. Our analysis considered the VSS-SF and DHI as outcomes, while the NPS, NRS, and HADS were treated as time-varying exposures. Both exposures and outcomes were measured across 3 repeated measures: baseline, 6 months, and 12 months. To properly handle correlated observations from the same subjects over time, we incorporated a random intercept for each subject in our models. We conducted both crude and adjusted analyses, with adjustments made for follow-up time and sex as categorical covariates and age as a continuous covariate.

To ensure the assumptions necessary for linear regression were met, we assessed the distribution of model residuals through histograms and qq-plots, confirming their approximate normal distribution. The associations between musculoskeletal pain (NPS and NRS) and dizziness (VSS-SF and DHI) were reported using regression coefficients for musculoskeletal pain, along with their corresponding 95% CI and *P*. In the context of linear mixed effects models, these regression coefficients capture both the within-subject effect (change in dizziness for a 1-unit change in pain within the same patient) and the between-subject effect (difference in dizziness for a 1-unit difference in pain between 2 patients), while holding the other covariates constant.

We further explored whether the association between pain and dizziness was modified by psychological distress (HADS). To explore this, we extended the above described linear mixed effects to include both the main effects and interaction terms of pain and HADS, along with the previously mentioned covariates. The model terms of pain and age were included as continuous, while HADS, time, and sex were included as categorical terms. HADS was categorized into 2 groups according to the total HADS cut-off score: no psychological distress <19 and psychological distress ≥19. To assess whether VSS-SF or DHI changed differently across HADS groups, we performed a likelihood ratio test by comparing models with and without the interaction term. The interaction analysis was also visualized in graphical formats.

Using mixed effects models, we also estimated *P* for trend in VSS-SF and DHI scores from baseline to 12-month follow-up for clinically relevant categories of pain levels. Follow-up time was incorporated as a continuous variable in models, whereas the pain variables (both NPS and NRS) were treated as categorical exposure terms with the number of pain sites categorized as 0 to 3, 4 to 6, and 7 to 10 and pain intensity categorized in accordance to the above mention cut-off values for severity. To enable the calculation of *P* for trend within each pain category, we further included an interaction term between follow-up time and the 2 pain variables in the models. The time-trend analyses were adjusted for sex and age.

Data was analyzed using the statistical software package Stata 17.0 (StataCorp LLC; College Station, Texas, USA).

### Ethics

The study was approved by the Regional Ethical Committee (REK 2019/6849) and the Norwegian Agency for Shared Services in Education and Research [Sikt] and further registered at ClinicalTrials.gov (no. NCT04241822; January 27, 2020) prior to the inclusion of patients. The study was committed to the criteria laid in the Declaration of Helsinki.^37^ The authors declare no conflict of interest.

### Role of the Funding Source

The funder played no role in this study’s design, conduct, or reporting.

## RESULTS

### Characteristics of the Participants

The response rate was 77.7% at 6 months and 74.3% at 12 months follow-up. Two responders were lost to follow-up due to invalid contact information. Characteristics of the study sample are listed in Table 1. The majority were female and the median duration of dizziness at baseline was 21.5 months (range = 3 months to >40 years). Almost 9 out of 10 responders reported dizziness during the preceding 6 months at 6-month and 12-month follow-ups. VSS-SF and DHI scores decreased at 6 months and stabilized toward 12 months. Yet, the symptom level was high (VSS-SF > 12), and the perception of disability was moderate (DHI > 30) at all measurement times (Table 1). NPS, NRS, and HADS changed little during the follow-up period.

**Table 1:** Characteristics of the Study Sample at Baseline, 6 Months, and 12 Months*^a^*

Characteristics	Baseline (n = 150)	6 mo (n = 114)	12 mo (n = 110)
Sex			
Male, n (%)	53 (35)	43 (38)	40 (36)
Female, n (%)	97 (65)	71 (62)	70 (64)
Age mean (SD)	46.5 (12.7)	–	–
Diagnostic categories (ICD-11), n (%)			
Episodic vestibular syndrome	97 (65)	–	–
Chronic vestibular syndrome	49 (33)	–	–
Other	4 (3)	–	–
Dizzy last 6 mo, n (%)	150 (100)	101 (88.6)	96 (87.3)
VSS-SF*^b^* mean (SD)	17.1 (9.5)	13.6 (9.8)	13.5 (10.5)
DHI*^b^* mean (SD)	38 (20.1)	31.4 (20.9)	30.6 (20.3)
NPS mean (SD)	4.5 (2.5)	4.3 (2.5)	4.0 (2.4)
NRS mean (SD)	4.0 (2.3)	3.7 (2.5)	3.7 (2.5)
HADS total mean (SD)	10.2 (7.5)	10.4 (7.9)	10.3 (7.9)
HADS anxiety	6.2 (4.4)	6.2 (4.7)	6.0 (4.8)
HADS depression	4.0 (3.8)	4.2 (3.7)	4.3 (3.7)
HADS ≥19, n (%)	19 (13.3)	16 (14.4)	18 (16.7)

^a^ DHI = dizziness handicap inventory; HADS = hospital anxiety and depression scale; ICD-11 = The International Classification of Diseases 11th revision; NPS = number of pain sites; NRS = numeric rating scale; VSS-SF = Vertigo symptom scale—short form.

^b^ Two patients did not respond to the VSS-SF, and 3 did not respond to the DHI at baseline.

Approximately 15% (range 13%–16%) of the participants were categorized with psychological distress (HADS ≥19; Table 1). This sub-group reported more severe dizziness and dizziness-related disability, more pain sites, and higher pain intensity compared to those with no psychological distress (HADS <19; Table 2).

**Table 2:** Average Score Across All Time Points in Dizziness Severity and Disability, Number of Pain Sites, and Pain Intensity According to the Level of Psychological Distress*^a^*

Variables	HADS <19	HADS ≥19
Outcome		
VSS-SF mean (SD)	13.7 (9.2)	22.4 (11.3)
DHI mean (SD)	30.7 (19.1)	52.1 (20.8)
Exposure		
NPS mean (SD)	4.1 (2.4)	5.1 (2.6)
NRS mean (SD)	3.7 (2.4)	4.5 (2.3)

^a^ DHI = dizziness handicap inventory; HADS = hospital anxiety and depression scale; NPS = number of pain sites; NRS = numeric rating scale; VSS-SF = vertigo symptom scale—short form.

#### Association of the Number of Pain Sites and Pain Intensity With Dizziness Severity and Dizziness-Related Disability

Regression analysis showed a positive association of NPS with VSS-SF (*P* < .001) and DHI (*P* < .001; Table 3). NRS was also associated with VSS-SF (P < .001) and DHI (*P* < .001). These associations remained after adjusting for time, sex, and age. The regression coefficient in Table 3 shows that for 1 unit increase in pain site VSS-SF increases by 0.84 points, and DHI increases by 1.54 points after adjusting for time, sex and age. Similarly, for 1 unit increase in pain intensity VSS-SF increases by 0.77 and DHI by 1.30 points. For a more detailed interpretation of these coefficients, see the description in the statistical section in methods.

**Table 3:** Association of the Number of Pain Sites and Pain Intensity With Dizziness Severity and Dizziness-Related Disability*^a^*

Variables	Crude Model Coefficient (95% CI)*^b^*	*P*	Adjusted Model*^c^* Coefficient (95% CI)	*P*
VSS-SF				
NPS	1.06 (0.65–1.46)	<.001	0.84 (0.45–1.23)	<.001
NRS	0.92 (0.55–1.30)	<.001	0.77 (0.41–1.13)	<.001
DHI				
NPS	1.99 (1.14–2.84)	<.001	1.54 (0.71–2.37)	<.001
NRS	1.63 (0.85–2.40)	<.001	1.30 (0.55–2.05)	.001

^a^ The coefficient quantifies the difference in dizziness for 1 unit difference in pain. DHI = dizziness handicap inventory; NPS = number of pain sites; NRS = numeric rating scale; VSS-SF = vertigo symptom scale—short form.

^b^ Estimated using linear mixed effect models for longitudinal data.

^c^ Adjusted for time, sex, and age.

#### Associations Stratified by Psychological Distress

**Figure 1:**
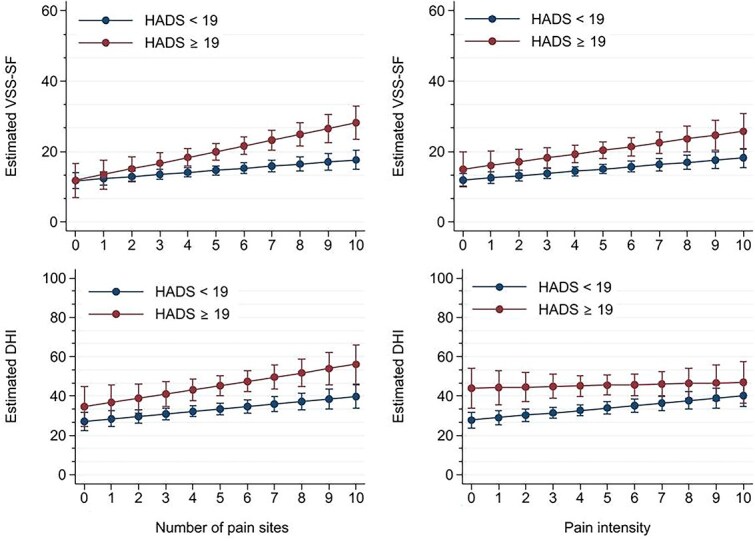
Change in the Association of VSS-SF and DHI With the Number of Pain Sites and Pain Intensity in Patients With and Without Psychological Distress According to HADS. DHI = dizziness handicap inventory; HADS = hospital anxiety and depression scale; VSS-SF = vertigo severity scale—short form.

Table 4 and Figure 1 show that the association between pain and dizziness-related outcomes is stronger in patients who also report psychological distress. According to the interaction analysis, the association between NPS and VSS-SF was stronger (*P =* .02) in the group with HADS ≥19, compared with the association in the group with HADS <19. The same pattern was observed with higher coefficients in the association of NRS with VSS-SF in addition to NPS with DHI, although the P for interaction were not significant. Table 4 also shows that for each unit increase in pain site or unit of pain intensity VSS-SFand DHI increased more (higher coefficient) in the group of patients reporting psychological distress (HADS ≥19) compared to those without psychological distress (HADS <19). This can be visually observed in Figure 1.

**Table 4:** Association Between the Number of Pain Sites and Pain Intensity With Dizziness Severity and Disability Modified by Psychological Distress*^a^*

Variables	HADS <19 Coefficient (95% CI)*^b^*	HADS ≥19 Coefficient (95% CI)*^b^*	*P* for Interaction*^c^*
VSS-SF			
NPS	0.59 (0.17–1.00)	1.64 (0.82–2.46)	.02
NRS	0.63 (0.25–1.01)	1.08 (0.21–1.95)	.35
DHI			
NPS	1.25 (0.36–2.14)	2.15 (0.43–3.88)	.34
NRS	1.25 (0.45–2.05)	0.31 (−1.53–2.14)	.34

^a^ Coefficient = regression coefficient; DHI = dizziness handicap inventory; NPS = number of pain sites; NRS = numeric rating scale; VSS-SF = vertigo symptom scale—short form.

^b^ Estimated using linear mixed effect models for longitudinal data with interaction terms and with adjustment for time, sex, and age.

c *P* for interaction was obtained by likelihood ratio test.

Table 5 shows the estimated decrease in VSS-SF and DHI over time within the categories of pain sites and pain intensity. The reduction of VSS-SF and DHI in the group with the lowest number of pain sites was not statistically significant. VSS-SF and DHI decreased significantly but were not clinically relevant (VSS-SF ≥ 12 and DHI ≥ 29) in patients with 4 or more pain sites. Regarding pain intensity, patients who scored 4 or higher on the NRS scale at baseline still had a severe level of dizziness (VSS-SF ≥ 12) and a moderate level of dizziness-related disability (DHI ≥ 29) 12 months later.

**Table 5:** Time Trends in Dizziness Severity (VSS-SF) and Dizziness-Related Disability (DHI) Across the Number of Pain Sites (NPS) and Across Levels of Pain Intensity (NRS)*^a^*

Outcome Variables	n	NPS 1-3*^b^* Mean (SD)	n	NPS 4–6 Mean (SD)	n	NPS 7–10 Mean (SD)	n	NRS 1-3*^c^* Mean (SD)	n	NRS 4–6 Mean (SD)	n	NRS 7–10 Mean (SD)
VSS-SF												
Baseline	31	12.5 (7.6)	64	15.5 (8.1)	46	21.7 (10.1)	60	13.7 (7.9)	58	19.3 (9.1)	22	19.4 (11.3)
6 mo	21	11.3 (9.8)	52	11.6 (8.4)	36	17.6 (10.9)	44	10.8 (9.0)	47	16.5 (11.1)	18	12.5 (6.4)
12 mo*^d^*	24	12.5 (10.4)	50	12.5 (10.5)	34	15.7 (10.5)	47	11.4 (9.3)	47	15.8 (11.8)	14	13.1 (8.5)
*P* for trend		.83		< .001		< .001		.02		.001		.007
DHI												
Baseline	31	33.5 (18.5)	62	35.5 (17.9)	47	44.2 (22.6)	31	34.0 (18.0)	62	40.3 (21.8)	47	42.7 (19.7)
6 mo	20	25.0 (23.2)	51	28.7 (18.0)	36	39.2 (22.0)	20	26.6 (20.5)	51	35.1 (22.4)	36	34.1 (16.9)
12 mo	24	29.8 (22.2)	51	28.6 (18.8)	32	34.8 (21.2)	24	27.0 (20.5)	51	35.2 (21.1)	32	28.8 (15.1)
*P* for trend*^d^*		.15		< .001		.005		.005		.015		.001

^a^ DHI = dizziness handicap inventory; NPS = number of pain sites; NRS = numeric rating scale; VSS-SF = vertigo symptom scale—short form.

^b^ NPS 1–3 = 1 to 3 pain sites reported, NPS 4–6 = 4 to 6 pain sites reported, NPS 7–10 = 7 to 10 pain sites reported.

^c^ NRS 1–3 = mild pain, NRS 4–6 = moderate pain, NRS 7–10 = severe pain.

d *P* for trend obtained from linear mixed effects models as described in the methods.

## DISCUSSION

The longitudinal associations between the number of pain sites and pain intensity with dizziness severity and dizziness-related disability were statistically significant, identifying musculoskeletal pain as a risk factor for poorer dizziness outcomes. These results support previous findings of strong associations between dizziness and pain in this population.^24^

The findings in the present study indicated an initial reduction in dizziness severity and disability scores within the first 6 months, probably due to advice and treatment provided during the consultation at the Balance Clinic at recruitment, with subsequent stabilization of the scores at 12 months. However, the mean level of dizziness was still severe (VSS-SF ≥ 12), and disability moderate (DHI > 30) in patients with 4 or more pain sites or 4 or higher pain intensity on the NRS scale. There was little change in the number of pain sites, pain intensity and psychological distress during the follow-up period.

The number of pain sites and severity of dizziness revealed a stronger association in patients with psychological distress (HADS ≥19) compared to those with no distress (HADS <19), meaning that an increase in the number of pain sites from baseline to 6 months leads to a higher increase in dizziness severity among those with psychological distress compared to those without. This may not be surprising as there is a well-known association between persistent dizziness and psychological distress.^12^^,^^14^ Additionally, psychological distress can affect the way patients perceive and react to discomfort. However, only about 15% of our sample reported psychological distress, according to HADS.^36^ The proportion reporting psychological distress remained stable throughout the follow-up period, which emphasizes that the observed changes in dizziness cannot be attributed to changes in HADS.

Stratified by HADS, we did not find statistical significance in the association between pain intensity and dizziness severity, but the coefficient and confidence interval imply a trend toward a stronger association in those with higher psychological distress. A similar trend was seen in the association between the number of pain sites and dizziness-related disability but not between the pain intensity and dizziness-related disability. The uneven number of respondents with and without psychological distress suggests that we should interpret the statistical *P*s with caution and rather emphasize the coefficient values and the trends observed in the scatterplots. The findings hold a clinical relevance, indicating that a higher number of pain sites could impact the functional level due to dizziness over time, especially in those with additional psychological distress, and should not be rejected despite non-significant *P*.

Several explanations can help explain why the number of pain sites and pain intensity have an impact on persistent dizziness and why this association appears to be stronger when psychological distress is present. Pain can restrict a person’s ability to move freely, and it can disrupt the somatosensory system by creating a conflict between expected and actual sensory information, disturbing how the body is perceived.^38^ The presence of psychological distress may be attributed to prolonged illness duration combined with fear of movement, which influences the patient’s ability to handle dizziness.^14^^,^^39^ Prolonged exposure to pain and/or dizziness can sensitize the nervous system, making it more sensitive to stimuli.^40^ Combined with psychological distress, it can lead to fear avoidance and increased stress symptoms. The vestibular system is a multisensory system integrating input from vestibular, visual, and somatosensory systems under the influence of the cerebellum. Further, there is an overlap in the brain regions responsible for processing information related to postural control, pain, and emotions.^40^ Dizziness, pain, and anxiety all share common features related to instinctive threat perceptions. These responses are rooted in neurological linkages in the amygdala, which is responsible for the fight-or-flight response.^41^ The responses are highly sensitive and have low specificity, meaning they can easily misinterpret the information.^38^ Considering dizziness as a “standalone” vestibular condition, detached from its broader context of bodily and psychological distress, can, therefore, be limiting and obstruct recovery. Recognizing pain as a risk factor for persistent dizziness, along with psychological distress in a patient-centered assessment, can be essential for promoting successful recovery and preventing prolonged issues.

Although the VSS-SF and DHI score values decreased in the total study sample during follow-up, the reduction may not reach a level deemed satisfactory or meaningful for the patients. The decrease observed during the first period could be a result of the information and advice provided during their appointments at the Balance Clinic. However, the effect diminished gradually toward 12 months. Adhering to these recommendations over time requires personal effort, and any stagnation in progress may also be influenced by lower compliance. However, the stabilization over time is in line with previous findings and underscores the challenge of achieving complete recovery when symptoms persist.^4^^,^^10^^,^^12^^,^^42^

The average number of pain sites in the study sample consistently exceeded that found in a Norwegian population study. Scores in the current study ranged from 4.5 at baseline to 4.0 one year later, whereas a Norwegian population study reported an average of 2.3.^43^ The number of pain sites has not previously been reported in populations experiencing persistent dizziness and the results indicate that patients with this condition have a higher number of pain sites compared to the Norwegian population. Given that the number of pain sites has been shown to exhibit a robust association with non-musculoskeletal symptoms (eg, dizziness), reduced general health, and the ability to predict future disability,^31^^,^^32^^,^^43^ as well as anxiety and depression,^44^ it appears to be a valuable and suitable outcome measure for assessing the complexity of persistent dizziness. In terms of pain intensity (ranging from 4.0 at baseline to 3.7 one year later), the findings were lower than those reported in a systematic review of patients with dizziness (mean = 6.1)^23^ but similar to that reported in the general population in Norway.^45^ The time trend for follow-up over 12 months shows that there is an indication to investigate and treat musculoskeletal pain when patients report 4 or more pain sites or pain intensity of 4 or more on the NRS scale because this group of patients still report severe dizziness and moderate dizziness-related disability 12 months later.

The majority of the study sample had an average total HADS score which placed them in the “non-case” category (total scale: mean 10, HADS-anxiety 6, HADS-depression 4; Stern, 2014), which is in line with other Norwegian studies on patients with persistent dizziness.^20^^,^^46–48^ Other studies across different vestibular diagnoses have, however, reported psychological distress in around 40%^42^ to 50%^49^ of their samples. The prevalence of psychological distress is higher among people with dizziness, but it appears that there is great variation within individuals and populations that have been investigated.

### Strengths and Limitations

To our knowledge, this is the first study to explore the association between musculoskeletal pain and dizziness to identify comorbid pain as a risk factor for dizziness outcomes in a longitudinal design. Achieving response rates of 77.7% and 74.3% in online surveys, as seen in this study, is considered solid.^50^ The study is unique by observing the natural progression in patients over a 12 month period. The Balance Clinic covers a large geographic area, enhancing the representativeness of the patients and the validity of the findings. We employed standardized questionnaires such as DHI, VSS-SF, and HADS. The widespread use of these instruments among patients with dizziness allows for comparison with other studies, adding to the significance of the results. However, these questionnaires may not capture the full spectrum of the patient’s experiences of dizziness and mental health, potentially missing relevant information. It is important to emphasize that HADS captures symptoms of anxiety and depression only, and other psychological factors such as catastrophizing or fear avoidance behavior were not assessed in the present study. In a previous study, we examined whether dizziness catastrophizing is associated with pain without finding a significant association.^24^

In observational studies, external factors beyond our control may influence the results. Although we controlled for sex and age in the regression analyses, unmeasured confounding factors may exist. A limitation of this study is that we do not have information on how patients adhered to the advice and exercises given during the consultation at the Balance Clinic. We also do not know which patients may have sought and received other treatments during the follow-up, and if so, what type, dosage and duration of such treatments. These are factors that could have influenced the findings of the study**.** Another limitation of the study is the insufficient information regarding the cause of pain reported by the patients, thereby impeding our ability to establish whether the type of pain holds significance in its impact on dizziness. This should be further explored in future research.

### Clinical Implications and Future Perspectives

The novel aspect of this study lies in its comprehensive evaluation of widespread pain and its investigation of whether pain can impact the outcome of persistent dizziness. While prior research on dizziness and pain has primarily concentrated on the neck and head region in this patient population, it is crucial to acknowledge that chronic pain often extends beyond a single body area. Hence, it is essential to consider the broad spectrum of musculoskeletal pain. Identifying that the number of pain sites is a risk factor for the outcome of dizziness suggests that pain should be included as an integral part of the standardized assessment of patients with vestibular disorders. Interrupting or preventing sensitization, where the nervous system becomes hypersensitive to stimuli, could be a key goal in preventing long-term conditions. Clinicians should recognize that psychological distress did not improve over time in the study sample. This indicates that there may be substantial room for improvement in better management of co-morbid distress in patients with persistent vestibular dizziness. Future research should explore whether pain management as a sole intervention has a modulating effect on dizziness or if pain management in combination with vestibular rehabilitation has a better effect than vestibular rehabilitation alone. Furthermore, it should be investigated whether the type of pain or specific musculoskeletal disorders are more prominent in the demonstrated association with dizziness. Qualitative research addressing personal experiences related to the relationship between pain, emotions, and dizziness could yield valuable insights into the intricate nature of persistent dizziness. Further, it would be interesting to examine the influence psychological distress has on the association between dizziness and pain in more detail.

## CONCLUSION

In a large longitudinal study, a robust association between the number of pain sites and pain intensity with dizziness severity and dizziness-related disability were found, indicating that musculoskeletal pain is a potential risk factor for poor dizziness outcomes. The impact of musculoskeletal pain on dizziness-related outcomes is even stronger in patients with comorbid psychological distress. The findings emphasize the importance of identifying pain, especially the number of pain sites, as a risk factor for poor outcomes of persistent dizziness. Clinicians should be aware of the importance of considering musculoskeletal pain when the number of pain sites exceeds 4 or pain intensity exceeds 4 on the NRS scale. Clinicians should also be aware that less improvement in dizziness over time can be expected in patients experiencing both combined psychological distress and multiple musculoskeletal pain sites.

## Supplementary Material

2023-0832_R2_Supplementary_Figure_pzaf001

## Data Availability

Data may be available from the corresponding author upon reasonable request. Any data shared will be anonymized to ensure the confidentiality of the study participants.

## CLINICAL TRIAL REGISTRATION

The study was registered at ClinicalTrials.gov (no. NCT04241822 on January 27, 2020 prior to the inclusion of patients. The study was committed to the criteria laid in the Declaration of Helsinki.^37^
