# Supplementary material for: Musculoskeletal Pain as a Risk Factor for Poor Dizziness Outcomes: A Longitudinal Study Among Patients With Persistent Vestibular Dizziness
Source: Phys Ther. 2025 Jan 13;105(4):pzaf001. doi: 10.1093/ptj/pzaf001 (PMC11997662; doi:10.1093/ptj/pzaf001)
Supplement: 2023-0832_R2_Supplementary_Figure_pzaf001 [file 2023-0832_r2_supplementary_figure_pzaf001.pdf]

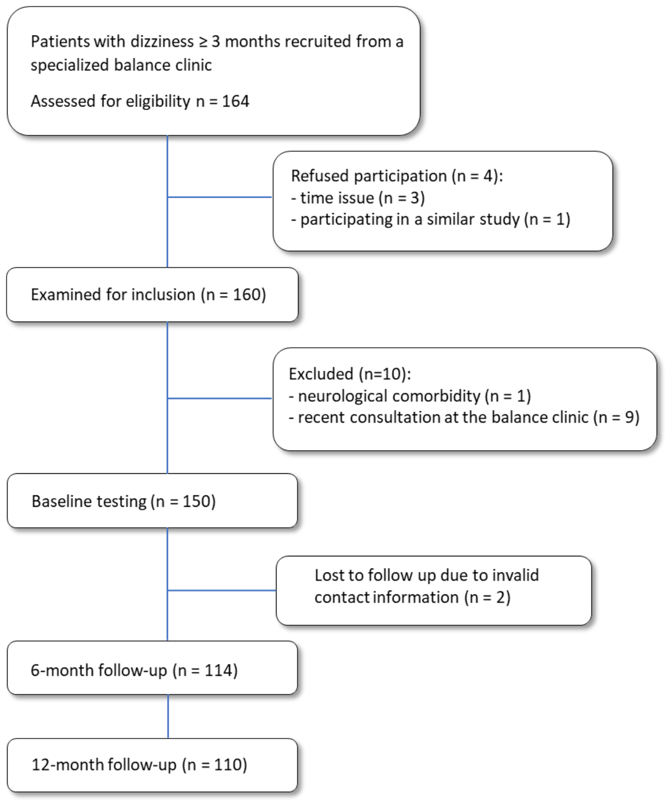

Supplementary Figure. Flowchart of the study design and the patients included in the 6- and 12-month follow-up study
